# Genetic Polymorphisms as Modifiers of Health Risks from Exposure to Toxic Elements: A Traditional Literature Review

**DOI:** 10.3390/toxics14050375

**Published:** 2026-04-27

**Authors:** Mariangela Palazzo, Andrea Borghini, Elisa Bustaffa, Silvia Baldacci, Francesca Gorini, Fabrizio Minichilli

**Affiliations:** Institute of Clinical Physiology, National Research Council, 56124 Pisa, Italy; mariangelapalazzo@cnr.it (M.P.); elisa.bustaffa@cnr.it (E.B.); silvia.baldacci@cnr.it (S.B.); francesca-gorini@cnr.it (F.G.)

**Keywords:** toxic elements, heavy metals, gene–environment interaction, single-nucleotide polymorphisms, cardiovascular disease, neurological disorders, cancer risk

## Abstract

A growing body of epidemiological and toxicological evidence indicates that exposure to toxic elements (TEs), including As, Cd, Cr(VI), Pb, and Hg, is associated with a wide range of adverse health outcomes, including cancer, neurological and cardiovascular diseases. Given their widespread presence and toxicity, understanding the factors underlying inter-individual differences in susceptibility is essential, as not all exposed individuals develop the same health effects. Genetic variability, particularly single-nucleotide polymorphisms (SNPs), is increasingly recognized as a key determinant of individual responses to TE exposure. Variants in genes involved in metal transport, detoxification, and DNA repair, including *DMT1*, *GSTP1*, *MT2A*, *hOGG1*, and *XRCC1*, may influence internal dose and biological effects and have been proposed as potential susceptibility markers. However, current evidence remains inconsistent due to small sample sizes, heterogeneous exposure assessments, and limited considerations of ethnic diversity and gene–environment interactions. Future research should prioritize large and well-characterized populations integrating detailed exposure and lifestyle data. This review focuses on genetic susceptibility and gene–environment interactions in TE exposure, with particular emphasis on SNPs as key modulators of individual risk. It summarizes major toxic metals, reviews epidemiological evidence of the associated health risks, and highlights the role of genetic background in modulating TE-induced toxicity.

## 1. Introduction

Potentially toxic elements (PTEs), or toxic elements (TEs), are environmental pollutants of growing scientific concern due to their impact on human health. They are often referred to as “heavy metals,” although this term is imprecise, as it may include metalloids (e.g., arsenic) and elements essential at trace levels [1]. Among TEs, arsenic (As), cadmium (Cd), hexavalent chromium (Cr(VI)), lead (Pb), and mercury (Hg) are the most relevant due to their high toxicity at low concentrations, environmental persistence, and bioaccumulation potential. These elements arise from both natural and anthropogenic sources and are widely distributed across air, water, soil, and food, leading to human exposure primarily through ingestion and inhalation, particularly in industrial and occupational settings [2,3]. Because they are non-biodegradable, toxic elements accumulate in environmental and biological systems, leading to long-term exposure and health risks. Epidemiological and toxicological evidence link As, Cd, Cr(VI), Pb, and Hg exposure to multiple adverse health outcomes affecting the nervous, renal, cardiovascular, immune, endocrine, and reproductive systems [4].

Given their pervasive nature and serious health risks, it is crucial to understand the factors that contribute to differences in susceptibility among individuals [5]. In fact, not all individuals exposed to similar levels of TEs develop the same health effects, reflecting substantial inter-individual variability.

A central mechanism underlying this phenomenon is gene–environment (G × E) interaction, whereby the effects of environmental exposures on disease risk are influenced by an individual’s genetic profile [6]. Among genetic determinants, single-nucleotide polymorphisms (SNPs) are the most common type of variation, present in a population with a frequency ≥ 1% [7]. They can be considered internal contributing factors in the susceptibility of individuals to TE effects. In this regard, SNPs are considered relevant as they belong to essential pathways, such as TE transport and detoxification, oxidative stress and DNA repair [8,9,10].

However, deeper investigations about mechanisms underlying the genetic influence on responses to toxic elements are needed and robust evidence about their potential role as disease predictors are required. Understanding the interplay between genetic factors and environmental exposures can be crucial for identifying individuals at a higher risk of adverse health outcomes, improving risk assessment, and informing preventive or therapeutic strategies. Therefore, this review focuses on genetic susceptibility and gene–environment interactions in the context of TE exposure. In particular, it examines the main toxic elements, summarizes epidemiological evidence of associated health risks, and highlights how genetic variability may modulate individual susceptibility to, or provide partial protection against, TE-related toxicity, based on the current state of the evidence. A bibliographic search was conducted in March 2026. The search was performed using PubMed, with keywords related to the topics mentioned above. To ensure comprehensive coverage, no restrictions were applied regarding publication type or study design. Only articles published in English were included, encompassing original research papers, reviews, and meta-analyses. The reference lists of the selected articles were also screened to identify additional relevant studies.

However, deeper investigations about mechanisms underlying the genetic influence on responses to toxic elements are needed and robust evidence about their potential role as disease predictors are required. Understanding the interplay between genetic factors and environmental exposures can be crucial for identifying individuals at a higher risk of adverse health outcomes, improving risk assessment, and informing preventive or therapeutic strategies. Therefore, this review focuses on genetic susceptibility and gene–environment interactions in the context of TE exposure. In particular, it examines the main toxic elements, summarizes epidemiological evidence of associated health risks, and highlights how genetic variability may modulate individual susceptibility to, or provide partial protection against, TE-related toxicity, based on the current state of the evidence. A bibliographic search was conducted in March 2026. The search was performed using PubMed, with keywords related to the topics mentioned above. To ensure comprehensive coverage, no restrictions were applied regarding publication type or study design. Only articles published in English were included, encompassing original research papers, reviews, and meta-analyses. The reference lists of the selected articles were also screened to identify additional relevant studies.

## 2. Sources of Toxic Elements and Routes of Exposure

Toxic elements (TEs) are naturally present in the Earth’s crust and are released into the atmosphere, hydrosphere, and lithosphere through processes such as rock weathering, volcanic activity, and geochemical cycling, establishing background levels that vary with local geology. However, over the past century, human activities—including industry, agriculture, and waste management—have significantly increased their mobilization and environmental distribution [11,12].

Arsenic (As) is naturally released through the weathering of As-bearing minerals and geothermal or volcanic activity and can persist in soils and sediments due to its past use in pesticides, feed additives, and wood preservatives. Major anthropogenic sources include mining and smelting, coal combustion, and industrial processes such as electronics and glass production, resulting in emissions to air, water, and waste streams [13]. Cadmium (Cd), naturally present in zinc, lead, and copper ores, is released through weathering and volcanic activity. Anthropogenic inputs are derived mainly from mining, smelting, battery production, wastewater discharge, and the use of Cd-contaminated fertilizers and pesticides, as well as the disposal or incineration of Cd-containing products such as batteries, plastics, pigments, and paints [14].

Hexavalent chromium (Cr(VI)) forms naturally through the oxidation of Cr(III)-bearing minerals, particularly in manganese-oxide-rich or ultramafic soils, but is predominantly associated with anthropogenic activities such as mining, electroplating, leather tanning, and pigment and dye production, as well as improper waste management, leading to the contamination of soils, water, and air [15,16]. Lead (Pb) occurs naturally in rocks and is released via weathering, forest fires, and particulates, but its environmental levels have increased more than 1000-fold in the past three centuries due to mining, smelting, battery production, the use of lead-based paints, fossil fuel combustion (including leaded gasoline), and lead–arsenate pesticides [17]. Mercury (Hg) is naturally emitted through volcanic activity, weathering, and ocean and soil emissions, but about two-thirds of atmospheric Hg is anthropogenic, mainly from coal combustion, mining, cement production, and metal manufacturing, which has significantly altered its natural cycle [18].

Human exposure to TEs occurs mainly through ingestion and inhalation, while dermal absorption is generally less relevant. Dietary intake is a major pathway: As is associated with rice and crops grown in contaminated soils [19]; Cd is found in vegetables, cereals, potatoes, and some fish [20]; Cr(VI) exposure occurs primarily via contaminated drinking water [21]; Pb is present in vegetables and drinking water, particularly from lead service lines [22,23]; and Hg exposure is mainly linked to fish and sea mammals [24].

Inhalation represents another major exposure route, especially in occupational settings. Elevated levels of As, Cd, and Pb have been observed in foundry workers [25], while Hg vapor exposure occurs in dental and industrial environments [24], and Cr(VI) exposure is common in chrome plating, welding, and surface treatment industries [26]. Nevertheless, inhalation exposure is also relevant in indoor environments, including homes, schools, offices, and public transport, where airborne TEs may pose non-carcinogenic and carcinogenic risks, often exceeding outdoor levels and international thresholds [27].

Smoking further increases exposure to TEs, as tobacco plants accumulate As, Cd, Cr, Hg, and Pb [28]. These elements are concentrated in different plant tissues, with Cd and Hg particularly present in leaves, and Cr, As, and Pb accumulating mainly in roots [29,30]. Tobacco smoke therefore exposes both active and passive smokers, with children being especially vulnerable due to higher exposure to contaminated household dust [31].

Dermal exposure is less significant but still possible: arsenite can penetrate the skin [32,33], Cd shows dermal bioaccessibility [34], Cr(VI) can be absorbed from contaminated soils and leather products [35], Pb may contribute to systemic burden in occupational settings [36], and Hg can be absorbed through the skin depending on chemical form and exposure conditions, including cosmetic use [37].

## 3. Biomonitoring and Health Effects of Toxic Elements

Given their widespread distribution and persistence in the environment, human exposure to TEs is frequent, making the evaluation of their health effects increasingly relevant. In recognition of their potential impact on public health, several TEs—particularly As, Cd, Pb, and Hg—were included among the top ten chemicals of major public health concern by the World Health Organization (WHO) [38]. To assess human exposure to these elements, biomonitoring approaches are widely used, measuring TE concentrations in accessible biological matrices through spectrometric techniques and subsequently evaluating exposure–disease relationships through epidemiological studies. Blood and urine represent the most used matrices for TE biomonitoring, although other matrices, including saliva, hair, nails, teeth, and breast milk, may also provide useful information, each presenting specific advantages and limitations [39]. The choice of matrix often depends on the specific element and the timing or form of exposure. For instance, As exposure is commonly assessed through urinary measurements [40], while Pb levels are typically determined in whole blood [41]. Cr (VI) is preferentially measured in the erythrocytic fraction [42], while Cd can be detected in blood as an indicator of recent exposure or in urine to reflect chronic exposure [43]. Similarly, Hg can be measured in both blood and urine depending on the chemical species involved: urine is more suitable for inorganic Hg forms (including elemental mercury and mercury vapor), whereas blood is the preferred matrix for methylmercury, the organic form mainly derived from fish consumption [44].

A growing body of epidemiological evidence has correlated TE concentrations in the human body with adverse health outcomes affecting multiple physiological systems, particularly cardiovascular, neurological, and oncological diseases, which significantly impact global morbidity, mortality, and quality of life [4]. Chronic exposure to toxic TEs, including As, Cd, Pb, Hg, and Cr(VI), is increasingly recognized as an environmental contributor to major non-communicable diseases. In the cardiovascular system, exposure to several TEs has been associated with an increased risk of cardiovascular diseases, particularly ischemic heart disease, the leading cause of mortality worldwide [45]. Meta-analyses indicate elevated risks associated with Pb, Cd, and As exposure, with more recent pooled analyses confirming increased cardiovascular disease risk for Hg as well [46], while evidence for Cr(VI) remains limited and inconsistent [47]. Neurological disorders represent another major health concern linked to TE exposure. Alzheimer’s disease (AD), the most common cause of dementia accounting for approximately 60–70% of cases, has been associated with chronic exposure to several TEs [48]. Epidemiological studies have reported higher Cd levels in AD patients and evidence linking Cd exposure to cognitive decline [49], while Hg and Pb have also been associated with cognitive impairment and increased dementia risk [50]. In addition, cumulative Pb exposure has been linked to significantly higher incidences of AD [51], while altered As metabolism patterns, elevated blood Cr levels, and Cr(VI)-induced neurotoxicity have also been implicated in neurodegenerative processes [52,53,54]. Importantly, TE exposure is not limited to adult health effects: prenatal or early-life exposure to Pb, Hg, and As has been associated with impaired neurodevelopment, including deficits in cognitive and motor functions as well as behavioral disorders [55].

Beyond cardiovascular and neurological outcomes, several TEs exhibit well-established carcinogenic potential. According to the International Agency for Research on Cancer (IARC), As, Cd, and Cr(VI) are classified as Group 1 human carcinogens, inorganic Pb compounds as Group 2A, and methylmercury as Group 2B. Epidemiological studies consistently associate As exposure with an increased risk of multiple malignancies, including lung, breast, liver, stomach, and hematological cancers [56]. Cd exposure has been linked to pancreatic [57], prostate [58], liver [59], renal [60], breast [47], and thyroid [61] cancers, while Pb exposure is associated with the increased incidence and mortality of several cancers, particularly those of the gastrointestinal and urinary tracts [62]. Hg has also been implicated in thyroid cancer risk [63], and Cr(VI) exposure has been associated with modest but significant increases in overall cancer incidence and mortality, particularly for respiratory and gastrointestinal cancers [64]. Overall, these findings highlight TEs as significant environmental risk factors contributing to the onset and progression of several chronic diseases.

## 4. Gene–Environment Interaction: The Role of Single-Nucleotide Polymorphisms in Susceptibility to Toxic-Element-Induced Effects

**Figure 1:**
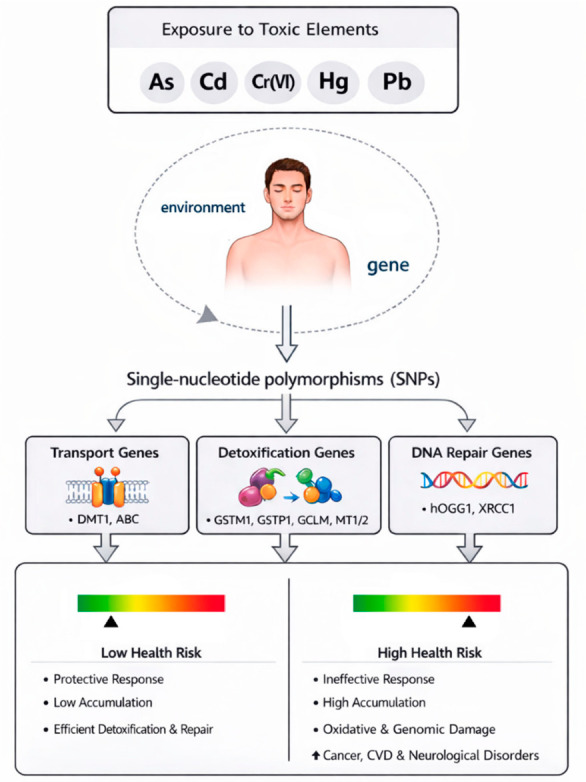
Gene–environment interaction in exposure to toxic elements: single-nucleotide polymorphisms can affect susceptibility to disease risk. The figure shows a schematic overview of gene–environment interactions in exposure to toxic elements, highlighting how single-nucleotide polymorphisms may modulate risk of individual susceptibility to disease. Abbreviations: As: arsenic; Cd: cadmium; Cr(VI): hexavalent chromium; Hg: mercury; Pb: lead; SNPs: single-nucleotide polymorphisms; DNA: deoxyribonucleic acid; CVD: cardiovascular disease. Image partially generated with AI Microsoft Copilot 365.

Multifactorial diseases arise from the interplay of genetic and environmental factors. These determinants act both independently and through gene–environment (G×E) interactions, whereby environmental exposures influence gene expression and genetic variations modulate individual susceptibility. In this context, genomic variability, particularly SNPs, contributes to inter-individual differences in response to environmental exposures [65]. SNPs affecting susceptibility to toxic elements can be functionally grouped into key pathways involved in metal transport, detoxification, and DNA repair, reflecting the complex nature of their metabolism and toxicity [8] (Figure 1). 

### 4.1. Transport

SNPs in genes encoding metal transporter proteins play an important role by directly influencing the uptake, distribution, and elimination of TEs.

Divalent Metal Transporter 1 (DMT1), encoded by *SLC11A2* located on chromosome 12q13 in humans, is a transmembrane protein that allows the cellular entry of some divalent cations, including Cd, Pb, Co, Mn, Ni, Zn, and Cu, into the cells. DMT1 is also referred to as DCT1 (Divalent Cation Transporter 1), NRAMP2 (Natural Resistance Associated Macrophage Protein 2), and SLC11A2 (Solute Carrier Family 11, member 2). In a study conducted by Kim et al., the relationship between the *DMT1* IVS4+44 C/A polymorphism and clinical parameters was investigated in 662 male Korean workers occupationally exposed to Pb. The findings suggested that individuals with the A/A genotype had an increased risk of lead-associated hypertension [66]. Moreover, from another study investigating the role of *SLC11A2* (*DMT1*) polymorphism rs224589 and enrolling 113 workers occupationally exposed to Pb, it emerged that individuals carrying the heterozygous CA genotype (54%) for *SLC11A2* showed higher blood Pb concentrations compared with both homozygous CC (wild-type) and AA (mutant) individuals. Furthermore, a significant inverse correlation was observed between blood Pb levels and hemoglobin exclusively in the CA subgroup, indicating a greater vulnerability to lead-related hematological alterations in correlation with this polymorphism [67].

Similarly, genetic variants of ATP-binding cassette transporters critically regulate the intracellular accumulation of TEs by mediating their transport outside from the cells. Polymorphisms in genes encoding for the ABC transporter can influence Hg accumulation and perinatal outcomes. Offspring carrying the *ABCC1* rs11075290 C allele have significantly higher cord blood Hg levels and present increased odds of small-for-gestational-age. Similarly, carriers of *ABCB1* rs2032582 GG genotype accumulate more mercury in cord blood and show a greater reduction in the mental development index [68].

### 4.2. Detoxification

Substantial evidence is reported about the contribution in TE toxicity exerted by SNPs of genes involved in detoxification processes, such as glutathione-related genes and metallothioneins, for their role in the binding and metabolism of TEs.

#### 4.2.1. Glutathione-Related Genes

Genes involved in the glutathione pathway are key mediators of detoxification processes, contributing to the conjugation and clearance of toxic compounds, while also counteracting oxidative stress. Genetic variants in these genes, including those encoding glutathione S-transferases (*GSTM1*, *GSTT1*, *GSTP1*) and the modifier (*GCLM*) and catalytic (*GCLC*) subunits of glutamate–cysteine ligase, can affect enzymatic function and glutathione biosynthesis, ultimately shaping inter-individual susceptibility to TE-induced toxicity. A study including a Chinese population of 850 subjects exposed to high levels of As in drinking water identified polymorphisms in glutathione-related genes *GSTO1* (rs11191979, rs2164624, rs4925), *GSTO2* (rs156697, rs2297235), and *PNP* (rs3790064) as associated with the increased risk of As-induced skin lesions in individuals exposed to high-dose inorganic arsenic, for impairment in the body’s ability to methylate and detoxify arsenic efficiently consequently to the presence of these variants [69].

*GSTP1* rs1695 polymorphism (AG + GG genotypes) is associated with an increased urinary percentage of inorganic arsenic (%InAs) and a decreased primary methylation index, indicating the reduced efficiency of these individuals in methylating inorganic As to its less toxic metabolites and increased susceptibility to its toxicity [70]. Altered methylation and reduction steps in As metabolism was confirmed by a recent genome-wide association analysis that reported how genetic variation in the flavin-containing monooxygenase and *GSTO* gene clusters significantly impacted blood and urinary levels of As metabolites [71].

Moreover, glutathione-related genes can play an important role in individual risk for arsenic-induced carotid atherosclerosis. *GSTT1* polymorphism was correlated with high urinary As levels and higher carotid intima-media thickness (IMT), evaluated as a marker of subclinical vascular damage in a cohort of Italian young adults exposed to environmental As [72].

Interestingly, polymorphisms can also be associated with protective effects. A meta-analysis including nine articles and 3324 subjects found that the *GSTM1* null genotype (rs4025935) was significantly associated with a lower susceptibility to As poisoning [73].

The presence of the SNPs of this pathway also contributes to the toxic effects observed in populations occupationally exposed to Cd. *CAT* rs7943316, *GSTP1* rs1695, the *GSTM1* null genotype, and the *GSTT1* null genotype in workers were linked to differences in the phenotypic expression of antioxidant enzymes, suggesting that individuals carrying these genotypes may be more susceptible to oxidative damage from Cd exposure [74].

Genetic polymorphisms in glutathione (GSH)-related genes influence Hg levels. Notably, carriers of the *GCLM* rs41303970 TT genotype exhibited lower Hg concentrations in both blood and hair compared with C-allele carriers, suggesting more efficient mercury elimination or reduced retention. Conversely, individuals with the *GSTM1* null genotype showed higher Hg, indicating greater accumulation of this TE [75].

*GSTP1* rs1695 and *CAT* rs1001179 substantially increase the risk of lung cancer in the context of elevated Cr exposure [76]. Toxic effects due to Hg exposure could be revealed in the presence of the *GCLC* rs1555903 C allele, as it correlates with a lower estimated glomerular filtration rate in non-exposed individuals and lower beta-2-microglobulin in exposed individuals, both markers of impaired renal function. Similarly, the combined effect of the *GSTA1* rs3957356 C and *GSS* rs3761144 G alleles is associated with higher urinary Hg levels in exposed individuals, suggesting that these variants may also contribute to increased metal retention and potentially greater toxicity. Conversely, the *GCLM* rs41303970 T allele is associated with protection against toxicity, as this SNP is linked to higher urinary Hg clearance, indicating enhanced elimination of this TE [77].

A cross-sectional study with 236 adults reported that *GCLC* rs17883901 was linked to enhanced antioxidant response, with higher concentrations of GSH as a function of Pb levels and *GCLM* rs41303970 exerting a protective effect against Pb accumulation, as carriers of at least one polymorphic allele for this gene had significantly lower blood and plasma Pb levels compared with those with the non-polymorphic genotype [78].

Genotyping of the *GSTM1*, *GSTT1*, and *GSTP1* genes was performed to investigate their potential association with heavy metal concentrations in 140 children exposed to Pb and Cd near an abandoned mining area in Kabwe, Zambia. The study found that the *GSTT1* null genotype was positively correlated with both blood Pb and Cd levels, while the *GSTP1* Ile/Val genotype (rs1695) was associated with a higher risk of Pb toxicity. This risk was even greater when these genetic variants co-occurred [79].

Furthermore, maternal polymorphisms in GSH-related genes can influence TE levels, with consequences on perinatal and birth outcomes. Pregnant women with *GSTM1-null*, *GSTT1-null*, and *GCLM* rs41303970 variants exhibit increased Hg accumulation and heightened oxidative stress, which were linked to an increased risk of preterm birth and reduced weight in offspring. Additionally, an increased frequency of children with a lower mental development index occurs when mothers carry the rare G allele of *GSTP1* rs1695. Moreover, increasing Hg exposure is associated with a lower psychomotor development index among *GCLC* rs761142 TT carriers [68].

#### 4.2.2. Metallothioneins

Metallothioneins (MTs) are cysteine-rich proteins that bind and sequester toxic elements, thereby reducing their bioavailability and protecting cells from metal-induced toxicity and oxidative stress [80]. Due to their abundant thiol groups, MTs bind biologically essential metals to help maintain metal homeostasis, as well as bind heavy metals to facilitate their transport and detoxification. Among all MT isoforms—including MT1, MT2, MT3, and MT4—the MT1A and MT2A subtypes are the predominantly expressed isoforms in humans.

A study enrolling 321 women revealed that SNPs of metallothionein 1A and 1B, *MT1A* rs8044719 and *MT1B* rs1599823, and *2A*, *MT2A* rs28366003 and *MT2A* rs10636, are associated with lower urinary Cd, indicating increased tissue retention and susceptibility to this TE [81]. In an analysis of 616 individuals, the *MT2A* gene polymorphism rs28366003 GG genotype was associated with significantly higher blood Cd and Pb levels compared with other genotypic subgroups [82], suggesting that individuals carrying the GG genotype may be more susceptible to metal toxicity and should take extra precautions to protect their health from the harmful effects of TEs.

*MT1A* gene polymorphisms rs11640851 and rs8052394 are associated with a negative correlation between creatinine-adjusted urine uric acid concentrations and cumulative blood Pb exposure, indicating that these genotypes may increase susceptibility to Pb-induced renal tubular dysfunction as reflected by uric acid excretion [83].

Regarding susceptibility to Hg exposure, the *MT1M* rs2270837 AA genotype and *MT2A* rs10636 CC genotype are associated with lower urinary Hg levels, while the *MT1A* rs8052394 GA and GG genotypes and *MT1M* rs9936741 TT genotype are associated with lower hair Hg levels [84]. Influence on Hg levels by SNPs of metallothioneins was subsequently demonstrated by another study with 165 women, in which *MT1M* rs9936741 was confirmed to be associated with significantly lower hair total Hg levels, suggesting a protective effect against accumulation, while *MT1M* rs2270836 was linked with higher hair Hg levels, indicating increased susceptibility to retention of this TE [85].

Overall, further investigations are needed on these genetic polymorphisms to better understand their impact on TEs and TE-induced adverse effects.

### 4.3. DNA Repair: Focus on the Base Excision Repair Pathway

Polymorphisms in DNA repair genes, including hOGG1 and XRCC1, contribute to inter-individual differences in disease susceptibility and cellular responses to DNA damage by altering the efficiency of the base excision repair pathway, which is involved in removing oxidative lesions and maintaining genomic stability.

The *hOGG1* Ser326Cys variant is associated with reduced enzymatic activity and impaired removal of oxidative DNA lesions, particularly 8-oxoguanine, leading to increased DNA damage accumulation and genomic instability. This polymorphism is linked to an elevated risk of cancer and other oxidative stress-related disorders, as well as altered sensitivities to chemotherapy and radiotherapy. Similarly, *XRCC1* variants such as Arg399Gln and Arg194Trp influence BER efficiency by modifying protein interactions within the repair complex. The Arg399Gln polymorphism is associated with decreased repair capacity and greater cancer risk, particularly under exposure to genotoxic agents, whereas the role of Arg194Trp appears less defined, with some evidence suggesting a potential protective effect. Overall, these variants may modulate individual susceptibility to environmental toxins and ionizing radiation [86].

*hOGG1* Ser326Cys (rs1052133) and *XRCC1* Arg399Gln (rs25487) polymorphisms can influence susceptibility to TEs. Borghini et al. reported that these polymorphisms in DNA repair genes are associated with increased susceptibility to As exposure in a cohort of 241 Italian young adults [87]. Carriers of the *hOGG1* Cys allele and the *XRCC1* Gln allele exhibited significantly shorter leukocyte telomere length in the context of higher urinary As levels, indicating enhanced genomic instability. These findings suggest that the combination of elevated As exposure and these DNA repair gene variants exacerbate telomeric DNA damage and may contribute to the development of arsenic-related health effects, highlighting a gene–environment interaction that increases individual vulnerability [87]. Moreover, *hOGG1* rs1052133 and rs159153 polymorphisms interact with As exposure and methylation capacity to increase the risk of urothelial carcinoma [88].

*hOGG1* rs1052133 and *XRCC1* rs25487 are frequently associated with increased DNA damage in workers exposed to Cd, indicating a reduced DNA repair capacity and greater susceptibility to genotoxic effects [9]. *hOGG1* rs1052133, along with other polymorphisms in DNA repair genes, *XPA* rs1800975, and *XPC* rs2228000 are also associated with the modulation of DNA instability biomarkers in workers occupationally exposed to Pb [89]. Moreover, individuals carrying *XRCC1* Arg399Gln (rs25487) and *hOGG1* Ser326Cys (rs1052133) polymorphisms exhibited increased DNA damage and oxidative stress when exposed to Hg [90].

Interestingly, the impact of *XRCC1* polymorphism on the genotoxicity exerted by the exposure to hexavalent chromium appears controversial. The *XRCC1* Arg399Gln (rs25487) homozygous variant genotype (Gln/Gln) is associated with increased chromosomal aberrations and greater susceptibility to chromosomal damage in workers exposed to this TE [91]. This result is in contrast with another study reporting that the *XRCC1* Arg399Gln (rs25487) variant is associated with reduced DNA damage in individuals occupationally exposed to Cr(VI), indicating a protective effect of this polymorphism against Cr(VI) toxicity [92]. This discrepancy likely reflects differences in the endpoints measured, population characteristics, or exposure levels. Other DNA repair polymorphisms, correlated with Cr effects, are *XPD* Lys751Gln (rs13181) and *XPC* Lys939Gln (rs2228000), which are associated with an increased susceptibility to lung cancer in individuals exposed to this element [93]. Moreover, the interaction between chromate exposure and the *XRCC3* rs2295152 T allele has a significant effect on micronuclei frequency, indicating a gene–environment interaction that increases susceptibility to chromate-induced genetic damage [94]. Best characterized SNPs are summarized in Table 1.

## 5. Conclusions and Future Perspectives: Key Points and Emerging Directions

TEs are bioaccumulative and exert a wide range of adverse effects on the human body, including cancer, neurological, and cardiovascular disorders. Their accumulation is influenced not only by environmental exposure levels but also by biological processes governing absorption, distribution, metabolism, and elimination. These processes vary substantially between individuals and strongly affect internal metal burden and toxicity outcomes. Investigating genetic variants and their interactions with environmental factors therefore provides crucial insights into individual susceptibility to TE-induced health risks.

It is now well established that genetic background represents a central component of the complex network of determinants defining sensitivity to metals. At the same time, additional internal and external factors, such as lifestyle, nutritional status, and co-exposures, further modulate biological responses. The interplay between genetic variability and environmental influences contributes to distinct phenotypes and heterogeneous responses to toxic exposures. Identifying variations in genes involved in TE metabolism remains essential for understanding susceptibility, identifying high-risk populations, and supporting the development of targeted preventive and therapeutic strategies. Among the most extensively studied candidate genes are those involved in transport, detoxification, and DNA repair, including DMT1, GSTP1, MT2A, hOGG1, and XRCC1. Various studies have explored the impact of SNPs within these genes, and some variants have been proposed as potential susceptibility markers for toxic element toxicity. Importantly, the overall risk should also be considered in terms of cumulative exposure across multiple pathways, which collectively influence the internal dose and biological effects of toxic elements.

However, findings remain inconsistent due to the heterogeneity of available studies. Differences in study design, sample size, exposure assessment methods, and population characteristics often hinder the integration of results and contribute to inconsistent findings. In particular, small cohorts and limited statistical power reduce the robustness of associations, while variabilities in exposure type (environmental vs. occupational) and intensity further complicate comparisons across studies. Moreover, the geographic distribution of research is imbalanced, with most evidence generated in specific regions, potentially introducing biases and limiting the generalizability of conclusions to underrepresented populations. In this context, the population-specific distribution of genetic variants warrants careful consideration. Certain alleles may be more prevalent in specific ethnic or geographic groups, and this can influence biological susceptibility to toxic elements and may partly explain inconsistencies across studies. At the same time, this raises important questions regarding the transferability of findings between populations, emphasizing the need for broader representation in genetic and epidemiological research.

Despite these limitations, the investigation of gene–environment interactions holds significant promise for practical applications and important questions regarding the transferability of findings between populations, emphasizing the need for broader representation in genetic and epidemiological research. Genetic variants associated with altered susceptibility could serve as biomarkers for identifying vulnerable individuals or subgroups, thereby improving risk stratification. Their integration into risk assessment frameworks may enhance the accuracy of exposure–response models and support the development of more effective prevention strategies. In the longer term, these insights could contribute to personalized and precision medicine approaches, where interventions are tailored according to individual genetic and environmental risk profiles.

To address current gaps, future research should prioritize multicentric study designs involving larger and more diverse populations exposed to varying levels of toxic elements. The harmonization of methodologies, including exposure assessments and biomarker measurements, is essential to improve comparability and reproducibility. The integration of epidemiological data with computational exposure models, alongside the application of artificial intelligence and machine learning, offers new opportunities to manage complex datasets and uncover subtle interaction patterns. Furthermore, the adoption of multi-omics approaches—encompassing genomics, epigenomics, transcriptomics, and exposomics—will enable a more comprehensive characterization of the exposome, capturing both internal biological responses and external environmental influences. This systems-level perspective is critical for elucidating the mechanisms underlying differential susceptibility.

Recent advances in induced pluripotent stem cell (iPSC) technology provide an additional complementary strategy. By reprogramming accessible somatic cells into iPSCs and differentiating them into tissue-specific models, researchers can investigate gene–environment interactions in a controlled and human-relevant context. These models allow the identification of susceptibility-associated variants, early molecular alterations, and potential therapeutic targets prior to disease onset, thereby supporting earlier and more effective prevention strategies [95,96]. The integration of approaches such as multicentric studies, advanced in vitro platforms, AI-driven data analysis, and multi-omics frameworks will help overcome current limitations and reduce uncertainty in the field. Ultimately, these efforts can improve risk assessment, strengthen prevention strategies, and facilitate the translation of mechanistic insights into public health policies and precision medicine applications.

Overall, this review underscores the role of gene–environment interactions and SNP-based susceptibility in shaping individual responses to toxic element exposure. Progress in this area will depend not only on methodological standardization and broader population representation but also on a stronger emphasis on translating scientific evidence into actionable tools for risk assessment, prevention, and personalized health management.

## Figures and Tables

**Table 1:** Best characterized single-nucleotide polymorphisms as promising markers of genetic susceptibility to TE exposure.

Toxic Element	SNP	Gene	Reference	Pathway	Effect	
As	rs11191979	GSTO1	[69]	Detoxification	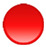	Impairment in body’s ability to methylate and detoxify As efficiently, ↑ risk of skin lesions
	rs2164624					
	rs4925					
	rs156697	GSTO2				
	rs2297235					
	rs3790064	PNP				
	rs1695 AG + GG genotypes	GSTP1	[70]		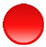	↓ Efficiency in methylating inorganic As, ↑ urinary percentage of inorganic AS, ↑ toxicity
	+ genotype	GSTT1	[72]		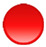	↑ Urinary As levels and ↑ carotid intima-media thickness
	rs4025935 null genotype	GSTM1	[73]		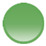	↓ As poisoning
	rs1052133 Ser326Cys	hOGG1	[87]	DNA repair	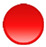	↑ Urinary As levels, ↓ leukocyte telomere length, ↑ genomic instability
	rs25487 Arg399Gln	XRCC1				
	rs1052133	hOGG1	[88]		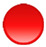	↑ Risk of urothelial carcinoma
	rs159153					
Cd	rs7943316	CAT	[74]	Detoxification	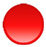	Altered expression of antioxidant enzymes, ↑ oxidative damage
	rs1695	GSTP1				
	null genotype	GSTM1				
	null genotype	GSTT1				
	null genotype	GSTT1	[79]		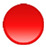	↑ Blood Cd levels
	rs8044719	MT1A	[81]		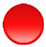	↑ Tissue Cd retention, ↓ urinary Cd
	rs1599823	MT1B				
	rs28366003	MT2A				
	rs10636					
	rs28366003 GG genotype	MT2A	[82]		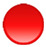	↑ Blood Cd levels
	rs1052133	hOGG1	[9]	DNA repair	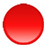	↓ DNA repair capacity, ↑ genotoxic effects
	rs25487	XRCC1				
Cr(VI)	rs1695	GSTP1	[76]	Detoxification	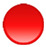	↑ Risk of lung cancer
	rs1001179	CAT				
	rs25487 Arg399Gln	XRCC1	[91]	DNA repair	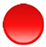	↑ Chromosomal aberrations and susceptibility to chromosomal damage
	rs25487 Arg399Gln	XRCC1	[92]		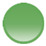	↓ DNA damage
	rs13181 Lys751Gln	XPD	[93]		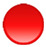	↑ Susceptibility to lung cancer
	rs2228000 Lys939Gln	XPC				
	rs2295152 T allele	XRCC3	[94]		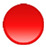	↑ Micronuclei frequency and genetic damage
Hg	rs11075290 C allele	ABCC1	[68]	Transport	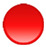	↑ Cord blood Hg levels and ↑ odds of small-for-gestational-age
	rs2032582 GG genotype	ABCB1			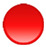	↑ Cord blood Hg levels and ↓ mental development index
	rs41303970 TT genotype	GCLM	[75]	Detoxification	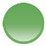	↓ Hg concentrations in blood and hair, ↑ efficient mercury elimination
	null genotype	GSTM1			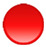	↑ Hg accumulation
	rs1555903 C allele	GCLC	[77]		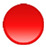	↓ Estimated glomerular filtration rate (eGFR) in non-exposed individuals and ↓ beta-2-microglobulin in exposed individuals, impairment of renal function
	rs3957356 C allele + rs3761144 G allele	GSTA1 + GSS			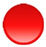	↑ Hg retention
	rs41303970 T allele	GCLM			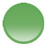	↑ Urinary Hg clearance, ↑ elimination
	null genotype	GSTM1	[68]		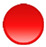	↑ Hg accumulation, ↑ oxidative stress, ↑ risk of preterm birth and ↓ weight in offspring
	null genotype	GSTT1				
	rs41303970	GCLM				
	rs1695 G allele	GSTP1			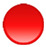	↓ Mental development index in offspring
	rs761142 TT genotype	GCLC			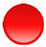	↓ Psychomotor development index
	rs2270837 AA genotype	MT1M	[84]		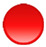	↓ Urinary Hg levels, ↑ Hg retention
	rs10636 CC genotype	MT2A				
	rs8052394 GA and GG genotypes	MT1A			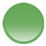	↓ Hair Hg levels
	rs9936741 TT genotype	MT1M				
	rs2270836	MT1M	[85]		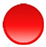	↑ Hair Hg levels
	rs9936741				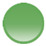	↓ Hair Hg levels
	rs25487 Arg399Gln	XRCC1	[90]	DNA repair	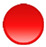	↑ DNA damage and oxidative stress
	rs1052133 Ser326Cys	hOGG1				
Pb	IVS4+44 C/A	SLC11A2	[66]	Transport	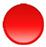	↑ Risk of hypertension
	rs224589 CA genotype	SLC11A2	[67]		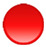	↑ Blood Pb concentration and ↑ hematological alterations
	rs17883901	GCLC	[78]	Detoxification	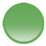	↑ Antioxidant response
	rs41303970	GCLM			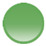	↓ Blood and plasma Pb levels
	null genotype	GSTT1	[79]		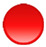	↑ Blood Pb levels
	rs1695 Ile/Val genotype	GSTP1			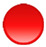	↑ Risk of Pb toxicity
	rs28366003 GG genotype	MT2A	[82]		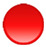	↑ Blood Pb levels
	rs11640851	MT1A	[83]		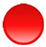	↓ Uric acid elimination, ↑ renal dysfunction
	rs8052394					
	rs1052133	hOGG1	[89]	DNA repair	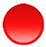	↑ DNA instability and genotoxicity biomarkers
	rs1800975	XPA				
	rs2228000	XPC				

Abbreviations: ↓: decrease of; ↑: increase of; As: arsenic; Cd: cadmium; Cr(VI): hexavalent chromium; Hg: mercury; Pb: lead; SNPs: single-nucleotide polymorphisms; DNA: deoxyribonucleic acid. Circles in the “Effect” column are green when SNPs are correlated with a protective action, red with increased toxic effects.

## Data Availability

No new data were created or analyzed in this study. Data sharing is not applicable to this article.

## Abbreviations

The following abbreviations are used in this manuscript:

AD	Alzheimer’s disease
As	Arsenic
Cd	Cadmium
Cr	Chromium
Hg	Mercury
IARC	International Agency for Research and Cancer
MTs	Metallothioneins
Pb	Lead
PTEs	Potentially toxic elements
PVC	Polyvinyl Chloride
SNPs	Single-nucleotide polymorphisms
TEs	Toxic elements
WHO	World Health Organization
